# Supplementary material for: A mobile intervention to reduce anxiety among university students, faculty, and staff: Mixed methods study on users’ experiences
Source: PLOS Digit Health. 2025 Jan 7;4(1):e0000601. doi: 10.1371/journal.pdig.0000601 (PMC11706487; doi:10.1371/journal.pdig.0000601)
Supplement: S1 Fig — (PDF) [file pdig.0000601.s001.pdf]

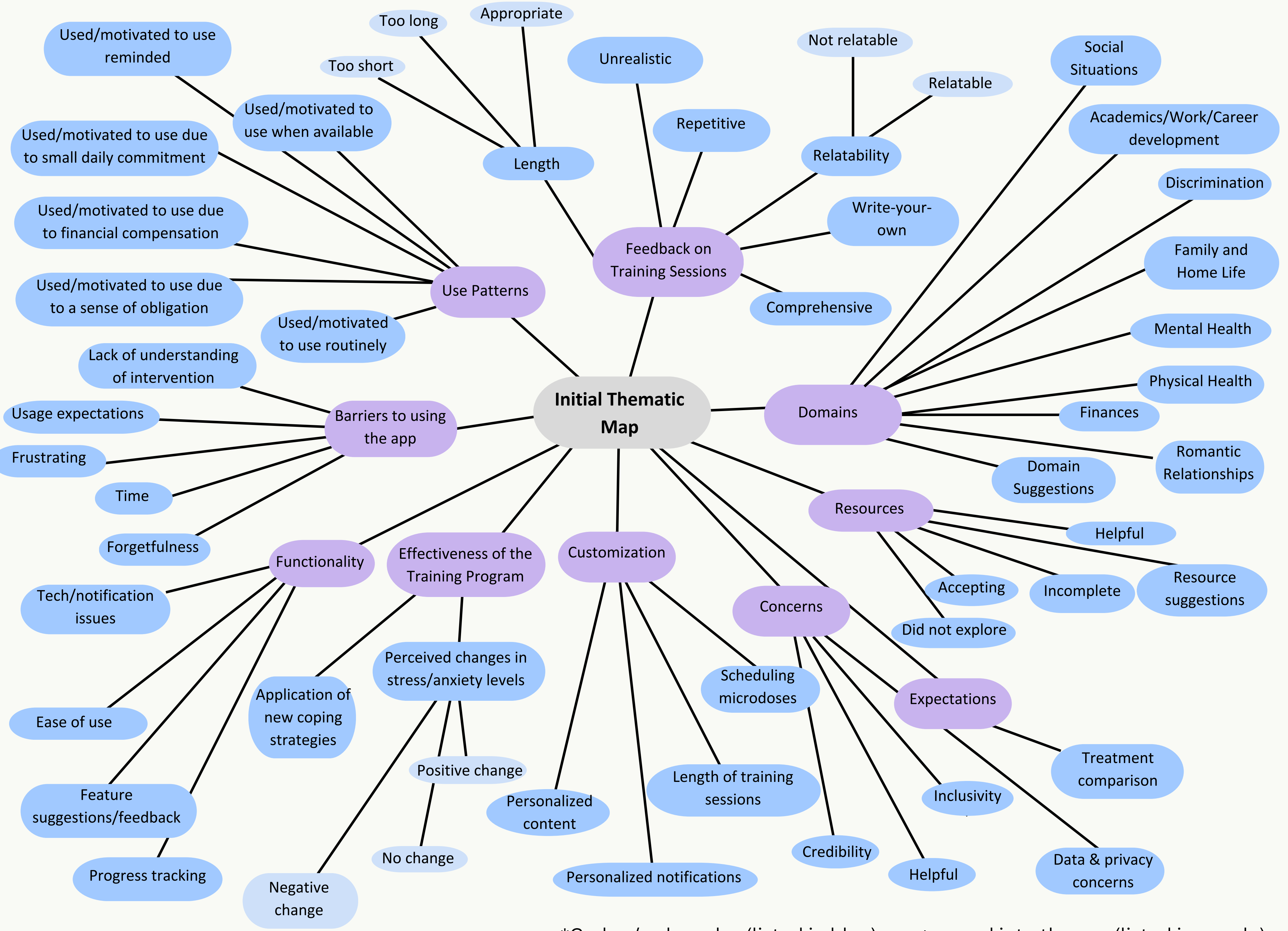

*Codes / sub-codes (listed in blue) are grouped into themes (listed in purple)

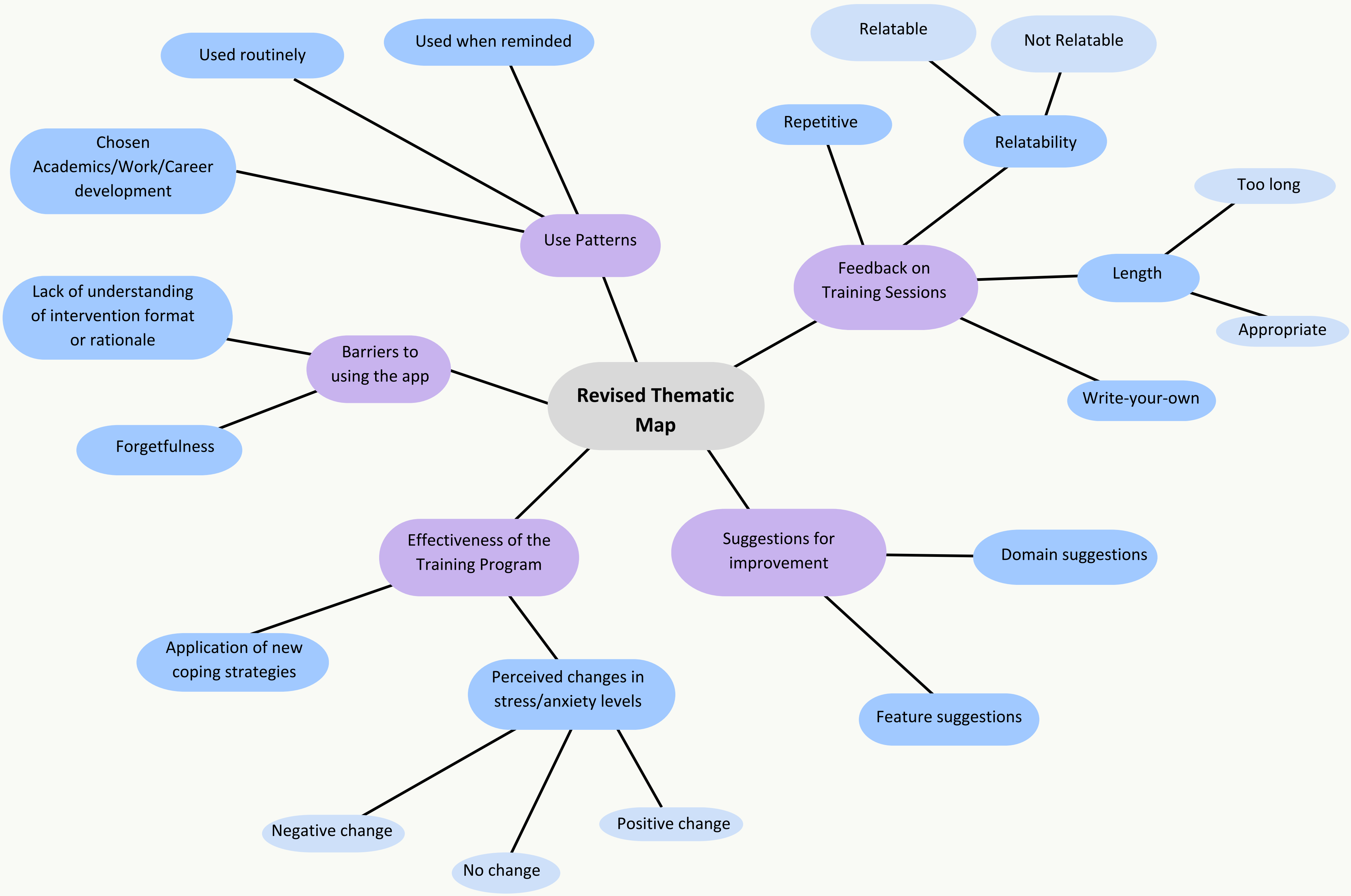

*Codes / sub-codes (listed in blue) are grouped into themes (listed in purple)
