# Supplementary material for: A mobile intervention to reduce anxiety among university students, faculty, and staff: Mixed methods study on users’ experiences
Source: PLOS Digit Health. 2025 Jan 7;4(1):e0000601. doi: 10.1371/journal.pdig.0000601 (PMC11706487; doi:10.1371/journal.pdig.0000601)
Supplement: S2 Text — (PDF) [file pdig.0000601.s003.pdf]

Hoos Think Calmly App Feedback Questions
IRB-SBS Protocol #4875

Introduce yourself

Consent process *(remind already signed & note 30-minute timeframe and $10 payment plan) (make sure to note Ps ID)*

Set audio recording *(see instructions below)*

Probe questions. *To be discussed during a semi-structured interview.*

We appreciate your participation in our research study. This program is designed to change negative interpretations (the part with brief stories relating to anxiety-provoking situations).

- What are your thoughts about the training task and the program?
    - What did you like? What did you dislike? What suggestions do you have for improvement?
    - Reactions to content of the stories? What would you like to see more or less of in the stories?
    - How helpful for reducing anxiety or stress about a specific situation in your life was this task?
    - Did the stories feel relevant to you? Why or why not?
    - Were you able to apply what you learned from these stories to specific stressful situations you were experiencing? Why or why not?

We will outline the resources we recommended within the app and ask their ideas about other resources that are needed.

- We offered the following materials and resources:
    - Brief stories designed to help people think about stressful situations differently
    - Tips and skills to help people manage feelings more effectively
    - Targeted recommendations for on- and off-campus resources (e.g., community groups, programming, career guidance, etc.) that may be helpful for the stress people are experiencing
    - Suggestions and resources for coping with experiences of discrimination
- What are your thoughts on these resources? What do you like and dislike about them?
- What are we missing? What other resources would be helpful to provide?

Finally, we will ask about how the intervention was implemented. We will also ask about the experience of using the app and whether they have any suggestions regarding improvements to the user interface and experience.

- What did you like about using the Hoos Think Calmly app? What didn't you like about it? What factors made using the app difficult or frustrating?
- What motivated you to use the program when you did use it? On days when you didn't use the app, if applicable, what got in the way of you using it? What would have made it easier for you to use the app?
- What did you think about the notifications sent by the app, both in terms of content and frequency? Did they help you remember to use the program? How could they be improved?
- How often did you use the program? How did you use the app (e.g., stress management, to enhance what you're learning through other treatment(s), etc.)?

- What did you think about the length of the training sessions (i.e., 5 minutes of training plus 2 minutes of assessments and resources)?

We may also share info about the different anxiety domains we targeted (e.g., anxiety about classes, social situations, health concerns, etc.) and ask whether we are missing important domains that trigger anxiety, and how well the identified domains are relatable and match user needs.

- The app targeted anxiety about the following broad domains:
    - *Social situations*
    - *Romantic relationships*
    - *Finances*
    - *Physical health*
    - *Mental health*
    - *Family and home life*
    - *Academics/Work/Career development*

- How relevant are these domains to your life? Do these feel like the most important domains?
- Which domains, if any, are we missing? Are there other major causes of anxiety/stress that are not captured by the domains listed above?
- Are there specific aspects of these domains that cause the most stress? If so, what are they?

Thank them for their feedback
