# Supplementary material for: A mobile intervention to reduce anxiety among university students, faculty, and staff: Mixed methods study on users’ experiences
Source: PLOS Digit Health. 2025 Jan 7;4(1):e0000601. doi: 10.1371/journal.pdig.0000601 (PMC11706487; doi:10.1371/journal.pdig.0000601)
Supplement: S1 Table — (DOCX) [file pdig.0000601.s004.docx]

**S1 Table: Results from Questionnaire by Group**

**Table A: How helpful do you find Hoos Think Calmly for reducing your anxiety?**

| **Group** | **1 =**  **Not at all** | **2 = Slightly** | **3 = Somewhat** | **4 =**  **Mostly** | **5 =**  **Very** | **Mean ± SD** |
| --- | --- | --- | --- | --- | --- | --- |
| Faculty | 5  (33.33%) | 7   (46.67%) | 2  (13.33%) | 1  (6.67%) | 0  (0.00%) | 1.93 ± 0.88 |
| Staff | 5  (6.75%) | 18 (24.32%) | 36  (48.65%) | 11  (14.86%) | 3  (4.05%) | 2.85 ± 0.91 |
| Graduate students | 8  (10.53%) | 18 (23.68%) | 35  (46.05%) | 13  (17.11%) | 2  (2.63%) | 2.78 ± 0.95 |
| Undergraduates | 17  (28.33%) | 14 (23.33%) | 24  (40.00%) | 5  (8.33%) | 0  (0.00%) | 2.28 ± 0.98 |

*Percentages do not add up to 100 when “Prefer not to answer” was endorsed

**Table B: How much could you relate to the stories presented during training sessions?**

| **Group** | **1 =**  **Not at all** | **2 = Slightly** | **3 = Somewhat** | **4 =**  **Mostly** | **5 =**  **Very** | **Mean ± SD** |
| --- | --- | --- | --- | --- | --- | --- |
| Faculty | 0  (0.00%) | 10 (76.92%) | 3   (23.07%) | 0  (0.00%) | 0  (0.00%) | 2.23 ± 0.44 |
| Staff | 2  (2.90%) | 14  (20.29%) | 29  (42.03%) | 16 (23.19%) | 7  (10.14%) | 3.18 ± 0.98 |
| Graduate students | 2  (2.82%) | 15 (21.13%) | 29  (40.85%) | 18 (25.35%) | 7  (9.86%) | 3.18 ± 0.98 |
| Undergraduates | 5  (8.77%) | 18 (31.58%) | 17  (29.82%) | 12 (21.05%) | 5  (8.77%) | 2.89 ± 1.11 |

*Percentages do not add up to 100 when “Prefer not to answer” was endorsed

**Table C: How believable did you find the stories’ endings in terms of those outcomes happening in your life?**

| **Group** | **1 =**  **Not at all** | **2 = Slightly** | **3 = Somewhat** | **4 =**  **Mostly** | **5 =**  **Very** | **Mean ± SD** |
| --- | --- | --- | --- | --- | --- | --- |
| Faculty | 0  (0.00%) | 10 (76.92%) | 1  (7.69%) | 1  (7.69%) | 0  (0.00%) | 2.25 ± 0.62 |
| Staff | 1  (1.45%) | 16 (23.19%) | 23  (33.33%) | 19 (27.54%) | 8  (11.59%) | 3.25 ± 1.01 |
| Graduate students | 1  (1.30%) | 9  (12.68%) | 32  (45.07%) | 22 (30.99%) | 7  (9.86%) | 3.35 ± 0.88 |
| Undergraduates | 6  (10.53%) | 17 (29.82%) | 13  (22.81%) | 18 (31.58%) | 3  (5.26%) | 2.91 ± 1.12 |

*Percentages do not add up to 100 when “Prefer not to answer” was endorsed

**Table D: I find the [length of the] training sessions to be:**

| **Group** | **1 =**  **Way too short** | **2 = Somewhat too short** | **3 = The right length** | **4 =**  **Somewhat too long** | **5 =**  **Way too long** | **Mean ± SD** |
| --- | --- | --- | --- | --- | --- | --- |
| Faculty | 0  (0.00%) | 1  (25.00%) | 0  (0.00%) | 1  (25.00%) | 1  (25.00%) | 3.67 ± 1.53 |
| Staff | 0  (0.00%) | 0  (0.00%) | 19 (61.29%) | 12  (38.71%) | 0  (0.00%) | 3.39 ± 0.50 |
| Graduate students | 0  (0.00%) | 0  (1.09%) | 17 (50.00%) | 13  (44.57%) | 0  (2.17%) | 3.43 ± 0.50 |
| Undergraduates | 0  (0.00%) | 0  (0.00%) | 10 (38.46%) | 15  (57.69%) | 1  (3.85%) | 3.65 ± 0.56 |

*Percentages do not add up to 100 when “Prefer not to answer” was endorsed
