# Supplementary material for: A mobile intervention to reduce anxiety among university students, faculty, and staff: Mixed methods study on users’ experiences
Source: PLOS Digit Health. 2025 Jan 7;4(1):e0000601. doi: 10.1371/journal.pdig.0000601 (PMC11706487; doi:10.1371/journal.pdig.0000601)
Supplement: Attachment — Submitted filename: Response to Reviewers.docx [file pdig.0000601.s005.docx]

Response to Reviewers

Reviewer #1:

**Comment 1:** The manuscript does a commendable job of analyzing the effectiveness of the Hoos Think Calmly app. However, there is a slight tendency to overestimate the intervention’s positive impact on anxiety reduction. Given that the majority of participants rated the app as only "somewhat helpful," a more balanced interpretation of these findings would strengthen the manuscript’s conclusions.

**Response 1:** Thank you for pointing this out. We have provided a more nuanced interpretation of the app's perceived effectiveness by further analyzing the quantitative and qualitative data across different demographic groups. This analysis led us to conclude that while graduate students were more likely to find the app effective, other groups, such as faculty, did not perceive the program as impactful. We believe this more nuanced interpretation is more meaningful, as it allows us to specifically target areas for improvement in future iterations of the app and provide more tangible recommendations for other DMHIs.

**Comment 2:** Similarly, the manuscript emphasizes user engagement, but the reliance on reminders and the issues with the notification system suggest that actual engagement might be lower than reported. Revising the discussion to reflect these nuances would provide a more accurate representation of user interaction with the app.

**Response 2:** Thank you for your suggestion. We have updated the Discussion to include comments regarding the notification system and its impact on user engagement:

*“Personal barriers to engaging with the app included individuals citing forgetfulness as a reason for not participating in the program. This issue is directly linked to the technical barrier of notification problems, which is particularly important given half of the interviewees reported struggling with forgetfulness. Many participants indicated that they used the program specifically when reminded, and appreciated the ability to schedule notifications at specific times. However, those who encountered issues with notifications stated that they only appeared when the app was open or running in the background. If the app was completely closed, reminders did not come through. This problem seemed specific to certain app versions and/or phone operating systems. Given that forgetfulness is a significant barrier to using the app, these technical issues likely impacted overall user engagement.” (p. 30-31)*

**Comment 3:** The issue of content relatability, particularly among older users and faculty, is significant. While this is acknowledged in the manuscript, its potential impact on the effectiveness of the intervention might be underemphasized. Strengthening the discussion around this point and offering more concrete suggestions for addressing this issue (e.g., tailoring content to different demographic groups) would be beneficial.

**Response 3:** Thank you for your feedback. We have revised the beginning of the Discussion to elaborate on the effectiveness and general acceptability of the program by demographic group.

*“Data was also analyzed by demographic/university role breakdown. A triangulation-based framework was adopted to compare the quantitative results with the corresponding qualitative data. When examining the perceived length of the program based on both quantitative and qualitative data, no demographic group disproportionately favored one response over the other groups. Instead, the general consensus was split between those who found the program to be the right length and those who considered it too long, as indicated in both the qualitative and quantitative findings. In regard to the program effectiveness, quantitative findings indicate that faculty tend to rate the program less positively than the other groups, finding it the least helpful, relatable, and believable. In contrast, staff and graduate students generally rate the program more positively, being more likely to find it helpful, relatable, and believable. Undergraduates fall somewhere in between (see S1 Tables). Qualitative data align with these quantitative findings, as all faculty interviewees described the program as unrelatable, and none reported an increase in flexible thinking skills. Meanwhile, the majority of graduate interviewees (8/10, 80%) found the scenarios relatable and noted an increase in flexible thinking skills. These findings suggest a relationship between the relatability of the scenarios and the perceived effectiveness of the program. Overall, both quantitative and qualitative results indicate that the current version of HTC may be best suited for graduate students, and further work is needed to improve the program for faculty.” (p. 28-29)*

With this background information now included, we have expanded our discussion on enhancing personalization for faculty members:

*“However, it is worth noting that all three faculty participants indicated that the study content was not relatable. They also rated the app as the least helpful in reducing anxiety on the user experience questionnaire compared to the other groups, which may be linked to their perception of the content (see S1 Tables). Therefore, we believe that increasing app personalization could be particularly beneficial for this demographic. Additionally, given that faculty members felt the content was tailored towards a younger audience, we strongly recommend involving more faculty in the co-design process for future iterations to ensure the content is relatable.” (p. 32)*

**Comment 4**: The technical barriers, particularly the inconsistency of push notifications, are briefly mentioned. However, these barriers are likely to have a more substantial impact on user engagement than currently reflected. A more detailed exploration of these technical challenges and their potential solutions would enhance the manuscript’s practical relevance.

**Response 4:** Thank you for your suggestion. We have revised the discussion to address the technical challenges experienced by participants. Please see response 2 above. We have also added a section to propose potential solutions:

*“To enhance notification customization and address both personal and technical barriers, we recommend offering users a range of notification delivery options, including push notifications, text messages, emails, or calendar reminders. Different notification strategies in DMHIs come with unique benefits and drawbacks that depend on user preferences. For instance, users with privacy concerns may prefer app notifications over text or email reminders, while those experiencing notification fatigue might opt for alternative systems, such as calendar integration. Given the pros and cons vary for each individual, it’s preferable to offer a range of options. Additionally, allowing users to choose the frequency of notifications may also be beneficial. One study found that college students receive an average of 260 notifications per day, reflecting their high level of digital engagement (Bakker et al., 2016). Despite this extensive digital exposure, research shows that younger internet users are less affected by stress, burnout, depression, and anxiety related to communication load compared to those over age 50 (Reinecke et al., 2016). Therefore, not only should notification types be customizable, but the frequency of notifications may also vary in effectiveness depending on age. Other research has shown that sending notifications containing tailored messages may also increase notification engagement (*Bidargaddi et al., 2018)*. In summary, expanding notification options and allowing users to choose what to opt into or out of will likely boost engagement and foster a sense of autonomy. However, with increased customization options, it’s essential to include recommended default settings at baseline (Bakker et al., 2016).” (p. 31)*

For reference, the citations are below:

Bakker D, Kazantzis N, Rickwood D, Rickard N. Mental health smartphone apps: Review and evidence-based recommendations for future developments. JMIR Ment Health [Internet]. 2016 Mar 1;3(1). Available from: <https://www.ncbi.nlm.nih.gov/pmc/articles/PMC4795320/>

Reinecke L, Aufenanger S, Beutel ME, Dreier M, Quiring O, Stark B, Müller KW. Digital stress over the life span: The effects of communication load and internet multitasking on perceived stress and psychological health impairments in a German probability sample. Media Psychol. 2016;20(1):90–115. doi: 10.1080/15213269.2015.1121832.

Bidargaddi N, Almirall D, Murphy S, Nahum-Shani I, Kovalcik M, Pituch T, et al. To prompt or not to prompt? A microrandomized trial of time-varying push notifications to increase proximal engagement with a mobile health app. JMIR Mhealth Uhealth. 2018 Nov 29;6(11)

**Comment 5:** While the methodological rigor is evident, the decision to code transcripts by a single coder after establishing high inter-coder reliability diverges from the original pre-registration. While this is understandable, a more transparent discussion of this change and its implications for the findings would be valuable.

**Response 5:** Thank you for this feedback. We have included additional comments regarding this decision and its implications for the findings within the methods section:

*“Due to prolonged ICR training, only two of the four trained coders were available for the official coding process. Given time constraints and the high ICR rating, deviations were made to the preregistration, with each transcript being coded by one of the two remaining coders instead of undergoing double coding. Based on norms in the literature, it is not uncommon for ICR to be calculated on a subset of data (10-25% of data units) to establish satisfactory reliability and then proceed to single coding (Burla et al., 2008; O'Connor, 2020). In line with this approach, we double coded 27% (6/22) of our transcripts before establishing ICR and moving to single coding. However, we acknowledge that single coding may introduce limitations in analysis due to potential inconsistencies in interpretation among independent coders, as well as increased bias from relying on a single analysis. To mitigate this, weekly meetings were held to address coding challenges and refine the codebook. The lead author refrained from coding to maintain an unbiased third-party perspective and acted as a moderator to help resolve disputes or clarify coding issues. In cases of disagreement, the lead author had the final say on coding decisions.” (p. 15)*

For reference, the citations is below:

O’Connor C, Joffe H. Intercoder reliability in qualitative research: Debates and practical guidelines. Int J Qualitative Methods. 2020;19. doi: 10.1177/1609406919899220.

Burla L, Knierim B, Barth J, Liewald K, Duetz M, Abel T. From text to codings: Intercoder reliability assessment in qualitative content analysis. Nurs Res. 2008;57(2):113–117. doi: 10.1097/01.NNR.0000313482.33917.7d.

**Comment 6:** It is noted that a considerable proportion of the references are self-citations related to your previous work on the MindTrails website and Cognitive Bias Modification for Interpretation (CBM-I). While it is reasonable to reference foundational studies, I encourage you to ensure that the manuscript is well-balanced by incorporating a wider range of studies from other researchers. This will not only broaden the context but also strengthen the manuscript’s engagement with the broader literature.

**Response 6:** Thank you for this suggestion. We have included the following references to strengthen the manuscript’s engagement with the broader literature:

1. Hoid D, Pan DN, Liao C, Li X. Effects of a smartphone-based, multisession interpretation-bias modification for anxiety: Positive intervention effects and low attrition. Int J Environ Res Public Health. 2023 Jan 27;20(3):2270. doi: 10.3390/ijerph20032270. PMID: 36767636; PMCID: PMC9915452.
2. Menne-Lothmann C, Viechtbauer W, Höhn P, Kasanova Z, Haller SP, Drukker M, van Os J, Wichers M, Lau JY. How to boost positive interpretations? A meta-analysis of the effectiveness of cognitive bias modification for interpretation. PLoS One. 2014;9(6). doi: 10.1371/journal.pone.0100925.
3. Yeung ES, Sharpe L. Cognitive bias modification for social anxiety: The differential impact of modifying attentional and/or interpretation bias. Cogn Ther Res. 2019;43:781–791. doi: 10.1007/s10608-019-10012-3.

Reviewer #2:

**Comment 1:** The sample size of 22 is a poor one to claim anything. Although I like the research methodology, which is sound and acceptable to me.

Please revise the paper with at least 500 samples to get something meaningful out of your work.

I also suggest to read these two articles:

a) Chattopadhyay S, Ray P, Land L, Li J. 'A Framework for Assessing ICT Preparedness for e-Health

Implementation', Accepted in IEEE 10th International Conference on e-Health Networking, Application

& Services", (IEEEHealthcom2008) to be held in Singapore from 7 – 9

th July (2008)

b)Chattopadhyay S., – “A Prototype Depression Screening Tool for Rural Healthcare: A Step towards

e-Health Informatics”, Journal of Medical Imaging and Health Informatics (2012); 2(3): 244-249.

If you feel these are insightful and helpful to your work to describe, you may please cite it.

Looking forward to receiving your revised version.

**Response 1:** Thank you for taking the time to read our paper and provide valuable feedback. We appreciate your suggested articles and have cited “A Prototype Depression Screening Tool for Rural Healthcare” to further highlight DMHIs' potential to increase access to mental health care.

*“DMHIs provide mental health support via technologies like web-based or mobile platforms, and have the potential to expand treatment delivery options and enhance access to mental health care, particularly in rural settings (Chattopadhyay, 2012).” (p. 3-4)*

Regarding our sample size, we would like to clarify that the decision to include 22 participants was based on established norms in the literature, which suggest that conducting around 15-30 qualitative interviews is standard for similar research goals (Berry, 2019; Gandy, 2024; Hennink, 2017; Marshall, 2015). Notably, we found that saturation was reached after 15 interviews, consistent with similar studies (Bucci et al., 2018; Hennink and Kaiser, 2022; Shkel, 2023; Smit et al., 2021). While we appreciate the interest in a larger sample, a sample size of 500 for qualitative interviews would not be feasible for this study and is not typical in comparable qualitative research.

For reference, the citations are below:

Berry N, Lobban F, Bucci S. A qualitative exploration of service user views about using digital health interventions for self-management in severe mental health problems. BMC Psychiatry. 2019 Jan 21;19(1).

Bucci S, Morris R, Berry K, Berry N, Haddock G, Barrowclough C, et al. Early psychosis service user views on digital technology: Qualitative analysis. JMIR Ment Health. 2018 Oct 31;5(4).

Gandy K. How many interviews or focus groups are enough? Eval J Australasia. 2024;24(3):211–223. doi: 10.1177/1035719X241266964.

Hennink M, Kaiser BN. Sample sizes for saturation in qualitative research: A systematic review of empirical tests. Soc Sci Med. 2022 Nov;292(1):1–10.

Hennink MM, Kaiser BN, Marconi VC. Code saturation versus meaning saturation: How many interviews are enough? Qual Health Res. 2017 Sep 26;27(4):591–608. Available from: <https://journals.sagepub.com/doi/full/10.1177/1049732316665344>

Marshall B, Cardon P, Poddar A, Fontenot R. Does sample size matter in qualitative research? A review of qualitative interviews in IS research. J Comput Inform Syst. 2013;54(1):11–22. doi: 10.1080/08874417.2013.11645667.

Shkel J, Green G, Le S, Kaveladze B, Marcotte V, Rushton K, Nguyen T, Schueller SM. Understanding users' experiences of a novel web-based cognitive behavioral therapy platform for depression and anxiety: Qualitative interviews from pilot trial participants. JMIR Form Res. 2023 Jun 20;7. doi: 10.2196/46062. PMID: 37338967; PMCID: PMC10337366.

Smit D, Vrijsen J, Groeneweg B, Vellinga-Dings A, Peelen J, Spijker J. A qualitative evaluation of a newly developed online peer support community for depression: Depression Connect (Preprint). J Med Internet Res. 2020 Nov 20;23(7).

Reviewer #3:

**Comment 1:** Provide specific statistical data on the prevalence of anxiety in college communities. Include recent, peer-reviewed sources to support this claim. This will establish a more compelling rationale for the study's importance.

**Response 1:** This is an excellent point. To give it the attention it deserves, we've included statistical data on the prevalence of anxiety in college communities in the Introduction, along with citations.

*“A 2023 study indicated that 41.8% of college students and 38.6% of faculty and staff had experienced symptoms consistent with severe or extremely severe anxiety over the past week (Meeks, 2023)” (p. 3)*

Meeks K, Peak AS, Dreihaus A. Depression, anxiety, and stress among students, faculty, and staff. J Am Coll Health. 2023;71(2):348–354. doi: 10.1080/07448481.2021.1891913.

**Comment 2:** Briefly explain what CBM-I entails and how it differs from other digital mental health interventions..

**Response 2:** Thank you for this feedback. We have included the following sentence to briefly explain CBM-I:

*“CBM-I training targets cognitive biases in threat perception, aiming to increase cognitive flexibility by reducing rigid negative thought patterns and encouraging more benign interpretations of ambiguous situations.” (p. 2)*

**Comment 3:** Specify the quantitative and qualitative methods used in the study. Explain how these methods complement each other and why this approach was chosen.

**Response 3:** The following sentence was added to better clarify the quantitative and qualitative methods used, as well as the rationale for choosing a mixed-methods approach:

*“This study used questionnaire and interview data to gather feedback from users of a mobile application called "Hoos Think Calmly" (HTC), which offers brief CBM-I training doses in response to stressors commonly experienced by students, faculty, and staff at a large public university. Mixed methods were used for triangulation to enhance the validity of the findings.” (p. 2)*

**Comment 4:** Provide more detail on how the thematic analysis was conducted. Mention the analytical framework used and how themes were identified and validated.

**Response 4**: Thank you for this suggestion. We have included additional details on how the thematic analysis was conducted:

*“Qualitative data was collected through semi-structured interviews from a subset of participants (n=22) and analyzed thematically using an inductive framework, revealing five main themes: Effectiveness of the Training Program; Feedback on Training Sessions; Barriers to Using the App; Use Patterns; and Suggestions for Improvement.” (p. 2)*

**Comment 5:** Include more specific data from the biweekly user experience questionnaires. Provide percentages or numerical data to support the statement about the program's helpfulness. This will add more substance to the quantitative findings.

**Response 5:** Thank you for the feedback. We have included specific data from the biweekly user experience questionnaires:

*“Additionally, biweekly user experience questionnaires sent to all participants in the active treatment condition (n=134) during the parent trial showed the most commonly endorsed response (by 43.30% of participants) was that the program was somewhat helpful in reducing or managing their anxiety or stress.” (p. 2)*

**Comment 6:** Expand on how the findings contribute to the broader field of digital mental health interventions. Explain how the results can inform future development and implementation of similar applications.

**Response 6:** Thank you for this suggestion. We have included details on how the findings may contribute to the broader development of DMHIs:

**“***Findings point to clear avenues to enhance the relatability and acceptability of DMHIs across diverse demographics through increased customization and personalization, which may help guide development of future DMHIs.” (p. 2)*

**Comment 7:** Strengthen the concluding statement by clearly articulating the main takeaways from the study and their significance for both research and practice in digital mental health interventions.

**Response 7:** Thank you for this suggestion. We have refined the concluding statement, and we believe the abstract is stronger due to your thoughtful feedback.

**Comment 8:** Expand the review of existing literature on digital mental health interventions (DMHIs) and Cognitive Bias Modification for Interpretation (CBM-I). Include more recent studies and meta-analyses to provide a comprehensive overview of the current state of research in this field. Critically analyze the findings of these studies to identify gaps in knowledge that your research aims to address.

**Response 8:** Thank you for the suggestion. We expanded the literature review, including recent studies and meta-analyses. Please refer to response 12 below for further expansion on the literature review.

*“A recent meta-analysis (Pauley et al., 2023) assessed the efficacy of digital interventions for various anxiety disorders, examining their impact against waitlist and standard care controls for specific disorders, and found positive effects on symptom reduction for DMHI’s for generalized anxiety, mixed anxiety, and social anxiety disorders. However, it was noted that further research is necessary to investigate which intervention formats have the greatest impact on treatment outcomes, particularly regarding the type and frequency of support provided...However, some studies report mixed results regarding the effectiveness of CBM-I (Cristea & Cuijpers, 2015; Hallion & Ruscio, 2011), highlighting the need for further investigation into the efficacy of these programs and how to improve their acceptability.” (p. 4)*

For reference, the citations are below:

Cristea IA, Kok RN, Cuijpers P. Efficacy of cognitive bias modification interventions in anxiety and depression: Meta-analysis. Br J Psychiatry. 2015;206(1):7–16. doi: 10.1192/bjp.bp.114.146761.

Hallion LS, Ruscio AM. A meta-analysis of the effect of cognitive bias modification on anxiety and depression. Psychol Bull. 2011;137(6):940–958. doi: 10.1037/a0024355.

Pauley D, Cuijpers P, Papola D, Miguel C, Karyotaki E. Two decades of digital interventions for anxiety disorders: A systematic review and meta-analysis of treatment effectiveness. Psychol Med. 2023;53(2):567–579. doi: 10.1017/S0033291721001999.

**Comment 9:** Develop a more robust theoretical framework that underpins the study. Clearly articulate the theoretical principles behind CBM-I and how they relate to anxiety reduction. Explain the mechanisms by which CBM-I is hypothesized to work and how these mechanisms are incorporated into the "Hoos Think Calmly" (HTC) application.

**Response 9:** Thank you for this suggestion. We have expanded on the theoretical principles behind CBM-I and hope this offers a stronger foundation for understanding the intervention's efficacy and theoretical basis:

*“The use of Cognitive Bias Modification for Interpretation (CBM-I) is a mechanistic intervention that may be an effective component of treatment for anxiety (Fodor, 2020;* Martinelli, 2022; Vrijsen, 2024)*, given extensive research showing that selective threat interpretations are common among individuals high in anxiety (*Murphy, 2007)*. CBM-I training is designed to modify threat-focused cognitive biases related to interpretation processes (i.e., assigning threatening meanings to ambiguous situations). “Hoos Think Calmly” (HTC) is a mobile application based on a specific CBM-I paradigm known as the ambiguous scenarios paradigm, which presents users with a series of ambiguous or mildly threatening scenarios (Mathews & Mackintosh, 2000). In the typical ambiguous scenarios format, the final word of each scenario appears as a word fragment that participants must complete. These fragments are typically designed to assign benign or neutral interpretations of the scenarios in most cases, helping to shift participants away from selectively negative or catastrophic thinking patterns. Through repeated practice and reinforcement, the goal is to help individuals develop more flexible interpretation patterns that are less rigidly negative, ultimately reducing anxiety.” (p. 4)*

For reference, the citations are below:

Fodor LA, Georgescu R, Cuijpers P, Szamoskozi Ş, David D, Furukawa TA, et al. Efficacy of cognitive bias modification interventions in anxiety and depressive disorders: A systematic review and network meta-analysis. Lancet Psychiatry. 2020 Jun;7(6):506–14.

Martinelli A, Grüll J, Baum C. Attention and interpretation cognitive bias change: A systematic review and meta-analysis of bias modification paradigms. Behav Res Ther. 2022 Aug;157:104180.

Mathews A, Mackintosh B. Induced emotional interpretation bias and anxiety. J Abnorm Psychol. 2000 Nov;109(4):602–15. PMID: 11195984.

Murphy R, Hirsch CR, Mathews A, Smith K, Clark DM. Facilitating a benign interpretation bias in a high socially anxious population. Behav Res Ther. 2007 Jul;45(7):1517–29. doi: 10.1016/j.brat.2007.01.007. Epub 2007 Feb 4. PMID: 17349970.

Vrijsen JN, Grafton B, Koster EHW, Lau J, Wittekind CE, Bar-Haim Y, et al. Towards implementation of cognitive bias modification in mental health care: State of the science, best practices, and ways forward. Behav Res Ther. 2024 Aug 1;179:104557. doi: 10.1016/j.brat.2024.104557. PMID: 38797055.

**Comment 10:** Provide a more detailed justification for conducting this specific study. Explain why evaluating user experience and acceptability is crucial for the development and improvement of DMHIs. Elaborate on how the findings from this study will contribute to advancing the field of digital mental health interventions.

**Response 10:** We have added a more detailed justification for this specific study to the paper:

***“****Additionally, a 2022 paper emphasized the importance of distinguishing between the feasibility/acceptability of a DMHI and its effectiveness. It’s argued that a program's efficacy cannot be accurately assessed if the intervention is not implemented properly and to its full potential (Park et al., 2022). Acceptability and feasibility studies help determine whether an intervention can meet users’ needs and can be implemented as intended. This is essential for evaluating user interest and satisfaction, willingness to engage with DMHIs, factors influencing participation, and reasons for continuing or dropping out—all of which are important for refining the intervention, especially in its early stages. The findings from this study will offer concrete strategies for improving DMHIs based on user feedback, which can be used to enhance the acceptability of future interventions.” (p. 6)*

For reference, the citation is below:

Park SY, Nicksic Sigmon C, Boeldt D. A framework for the implementation of digital mental health interventions: The importance of feasibility and acceptability research. Cureus. 2022 Sep 19;14(9). doi: 10.7759/cureus.29329. PMID: 36277565; PMCID: PMC9580609.

**Comment 11:** Offer a more comprehensive description of the HTC application. Include details about its design, functionality, and how it delivers CBM-I training. Explain how the app's features address the specific needs and challenges of the university community.

**Response 11:** Thank you for your feedback. We have included a more detailed description of the app in the introduction. We provide further elaboration on the design and functionality of the intervention in the Methods section. In response to how we addressed the specific needs and challenges of the university community, we conducted a co-design research process prior to implementing the HTC application. Our research team developed a preliminary design plan for HTC, incorporating feedback from users of an earlier version of MindTrails (the parent program). We then conducted beta testing sessions with various student groups and organizations in the university community where they learned about the program, tested the app, and answered questions about the program in a semi-structured interview. Feedback from these interviews was analyzed, enabling our team to identify key features important to the university community and incorporate relevant university-related stressors into the training scenarios. More information related to the co-design process can be found here <https://osf.io/preprints/psyarxiv/sjfbe>. To address your comments in the paper, we have included the following:

*“Hoos Think Calmly” (HTC) is a mobile application based on a specific CBM-I paradigm known as the ambiguous scenarios paradigm, which presents users with a series of ambiguous or mildly threatening scenarios (Mathews & Mackintosh, 2000). In the typical ambiguous scenarios format, the final word of each scenario appears as a word fragment that participants must complete. These fragments are typically designed to assign benign or neutral interpretations of the scenarios in most cases, helping to shift participants away from selectively negative or catastrophic thinking patterns. Through repeated practice and reinforcement, the goal is to help individuals develop more flexible interpretation patterns that are less rigidly negative, ultimately reducing anxiety. HTC offers these CBM-I training scenarios in brief 5-10 minute training sessions that are designed to increase flexible thinking in response to stressors commonly experienced by students, faculty, and staff at a large public university. To meet the specific needs of the university community, we engaged in outreach with student groups and organizations to beta test the app, review stressor domains, and develop scenarios tailored to their needs. We also consulted with leadership from local university programs for recommendations on in-app resources to incorporate.” (p. 4-5)*

**Comment 12:** Include more recent references to ensure the literature review reflects the most current research in the field. Ensure all claims are properly supported by credible, peer-reviewed sources. Consider including a mix of seminal works and cutting-edge research to provide a comprehensive overview of the topic.

**Response 12:** Thank you for the suggestion. We have updated the literature review with recent peer-reviewed studies.

*“Other studies have evaluated the acceptability of DMHIs through qualitative methods and have shown mixed feedback regarding attitudes toward and engagement with these tools. One study identified negative attitudes toward a Cognitive Behavioral Therapy (CBT) DMHI as users specifically found it to be inflexible, unrelatable, and hard to engage with (Venning et al., 2021). Another study revealed that participants had a positive attitude toward and experienced benefits from a DMHI, but the need to further personalize and creatively engage users to promote long-term adherence was emphasized (Rusch et al., 2022). When it comes to enhancing user motivation, engagement, and enjoyment, recent research indicates that games and gamification, when combined with CBT, have produced encouraging results (Amer et al., 2024; Salemink et al., 2022). As the mHealth landscape continues to evolve to meet user needs, ongoing evaluation of DMHIs is essential to keep pace with the advancements in the field.” (p. 5-6)*

For reference, the citations are below:

Amer NA, Abdelrazek S, Eladrosy W, et al. Computer-based cognitive behavioral therapy intervention for depression, anxiety, and stress disorders: A systematic review. J Cogn Ther. 2024;17:885–918. doi: 10.1007/s41811-024-00225-0.

Rusch A, Carley I, Badola P, Liebrecht C, McInnis M, Ryan KA, Smith SN. Digital mental health interventions for chronic serious mental illness: Findings from a qualitative study on usability and scale-up of the Life Goals app for bipolar disorder. Front Digit Health. 2022;4:1033618. doi: 10.3389/fdgth.2022.1033618.

Salemink E, de Jong SRC, Notebaert L, MacLeod C, Van Bockstaele B. Gamification of cognitive bias modification for interpretations in anxiety increases training engagement and enjoyment. J Behav Ther Exp Psychiatry. 2022;76:101727. doi: 10.1016/j.jbtep.2022.101727.

Venning A, Herd MC, Oswald TK, et al. Exploring the acceptability of a digital mental health platform incorporating a virtual coach: The good, the bad, and the opportunities. Health Informatics J. 2021;27(1). doi: 10.1177/1460458221994873.

**Comment 13:** Expand on the ethical considerations section. Provide more details about the informed consent process, including how participants were informed about the study's purposes, risks, and benefits. Elaborate on measures taken to protect participant confidentiality and data security, particularly given the sensitive nature of mental health data.

**Response 13:** The study's purposes, risks, and benefits were clearly outlined in the consent form. Interested participants read the consent form online and provided an e-signature via DocuSign to consent to participation. It was emphasized that participants have the right to withdraw from the study at any time, and we provided contact information for the research team in case they had questions before or after signing the consent form. To protect participant confidentiality, each participant was assigned a unique ID, and all data collected was linked to this ID. A list connecting codes to personal identifiers was stored in a password-protected document accessible only to the research team. The questionnaire data collected through the smartphone application contained no personally identifiable information and was associated solely with the participant's unique ID. Only the investigators have access to this data. Audio and video recordings from interviews were labeled with the participant's unique ID and temporarily saved in a shared folder accessible only to the research team. After the interviews were transcribed (and identifying information in the transcripts was removed) and fully reviewed, the recordings were deleted.

To address this in the paper, the “Ethics approval and consent to participate” section of the paper has been edited to the following:

*“This study was approved by the Institutional Review Board for Social and Behavioral Sciences (IRB-SBS) at the University of Virginia. All participants provided written informed consent via an electronic consent form that outlined the study’s purpose, risks, and benefits prior to study enrollment. To ensure confidentiality, each participant was assigned a unique ID after enrolling, and all data—including questionnaire responses and audio/video recordings—were securely stored and linked only to this ID, with access restricted to the research team.” (p. 6-7)*

**Comment 14:** Offer a more detailed explanation of the CBM-I training scenarios. Include information about how the scenarios were developed and validated. Discuss any pilot testing or iterations of the scenarios that occurred before the main study.

**Response 14:** Thank you for giving us the opportunity to clarify the development and testing of the CBM-I training scenarios. We have added the following to the Method section:

*“In the present study, some CBM-I scenarios were adapted from our existing pool of scenarios developed for previous MindTrails studies (Eberle et al., 2024; Ji et al., 2021; Larrazábal et al., 2024) and others were generated for the HTC application. Existing scenarios were adapted as needed to be relevant to university community members with different roles on campus and at different developmental stages. For example, a scenario pertaining to anxiety about handing in a paper to a teaching assistant might be kept the same (i.e., not adapted) for an undergraduate student, but modified to be focused on submitting a paper to an academic journal (graduate students) or giving an important report to a supervisor (staff members). Our goal was to preserve the core anxiety-provoking ambiguous stimulus for a scenario across groups but tailor the content to be more role-specific. This tailoring was guided in part by our qualitative interviews with university community members as well as in consultation with undergraduate students, graduate students, and faculty members on the research team who have lived experience as members of these groups. New scenarios were generated based on feedback from the qualitative interviews about topics identified as being particularly relevant for the university community. For example, we learned in feedback that many undergraduate and graduate students experience imposter syndrome and that this is a common source of stress and anxiety. We did not have any scenarios specifically pertaining to imposter syndrome in our existing pool, so we developed new scenarios focused on this topic. Scenarios were initially adapted and/or generated by a team of undergraduate research assistants who received training in how to write effective CBM-I scenarios. Subsequently, the HTC application was pilot tested, and scenarios were reviewed by other undergraduate students as well as graduate students and faculty in clinical psychology for small errors (e.g., typos) as well as for their effectiveness as CBM-I scenarios (i.e., whether the scenario vividly introduced a potentially threatening ambiguous situation and resolved that ambiguity in a positive or negative manner).” (p. 9-10)*

For reference, the citations are below:

Eberle JW, Daniel KE, Baee S, Behan HC, Silverman AL, Calicho-Mamani C, et al. Web-based interpretation training to reduce anxiety: A sequential, multiple-assignment randomized trial. J Consult Clin Psychol. 2024;92(6):367–384. doi: 10.1037/ccp0000896.

Ji JL, Baee S, Zhang D, Calicho-Mamani CP, Meyer MJ, Funk D, et al. Multi-session online interpretation bias training for anxiety in a community sample. Behav Res Ther. 2021;142:103864.

Larrazabal MA, Eberle JW, Vela A, Mehdi Boukhechba, Funk DH, Barnes LE, et al. Online cognitive bias modification for interpretation to reduce anxious thinking during the COVID-19 pandemic. Behav Res Ther. 2024 Feb 1;173:104463.

**Comment 15:** Offer more details about the semi-structured interview process. Describe how the interview guide was developed and piloted.

**Response 15:** Thank you for this question. We have added more details about the development of the semi-structured interview below and in the Method section of the manuscript.

*“The semi-structured interview consisted of a mix of open-ended questions about user perceptions of and suggestions for the application, and more targeted questions that addressed topics that would be relevant for app design and content decisions. Questions were based in part on prior qualitative interviews conducted by our team and common user experience measurement tools (Leventhal et al., in press). In prior qualitative interviews, the semi-structured interview guide was iteratively updated to prioritize questions related to different design decisions (e.g., earlier interviews focused on topics that should be covered in CBM-I scenarios, whereas later interviews focused more on developing a feasible implementation plan with respect to app notifications, session length, etc.).” (p. 11)*

For reference, the citations is below:

Leventhal EL, Toner ER, Davidson B, et al. Adapting a mobile anxiety intervention for a university community: Insights from a qualitative analysis. J Technol Behav Sci. 2024. doi: 10.1007/s41347-024-00445-3.

**Comment 16:** Provide a more detailed account of the thematic analysis process. Explain how themes were identified, refined, and validated. Include information about how disagreements between coders were resolved and how the final themes were decided upon.

**Response 16:** Thank you for your feedback. We have added the following comments throughout the Analysis section to provide a more detailed overview of the thematic analysis process:

*“We followed an inductive approach of open coding to allow for unexpected, data-driven themes to emerge (Roberts et al., 2019). As codes were created, they were grouped with relevant codes to highlight similarities and begin developing overarching themes. These themes were identified and agreed upon through group consensus, although they continued to be refined as codes were added and removed throughout the analysis process…The lead author refrained from coding to maintain an unbiased third-party perspective and acted as a moderator to help resolve disputes or clarify coding issues. In cases of disagreement, the lead author had the final say on coding decisions…Once coding was complete, the themes were reviewed and refined one final time by visualizing their relationships with the codes and with each other through thematic mapping” (p. 14-15)*

For reference, the citation is below:

Roberts K, Dowell A, Nie JB. Attempting rigour and replicability in thematic analysis of qualitative research data: A case study of codebook development. BMC Med Res Methodol. 2019 Mar 28;19(1):1–8.

**Comment 17:** Expand on the analysis of the survey data. Describe any statistical software used and provide more details about the specific descriptive statistics calculated. Explain how missing data, if any, was handled.

**Response 17:** Analyses were conducted in R Version 4.2.3 using the *mean()*, *sd(), table(), and prop.table()* functions in the base R package. We calculated the means and standard deviations for each survey question of interest across all weeks, despite the fact that some questions were asked on multiple biweekly surveys, because we were mainly interested in the overall ratings on each survey question and how they do or do not match responses to similar qualitative interview questions. Missing data here indicates that participants did not fill out the biweekly survey or skipped the question; we examined means and standard deviations using all available responses.

**Comment 18:** Provide more information about the psychometric properties of the measures used (OASIS, PHQ-2, PSS-4). Include details about their validity and reliability in similar populations.

**Response 18:** Thank you for this feedback. We have provided more information about the psychometric properties of the measures used.

*“Patient Health Questionnaire-2 (PHQ-2) was used to assess depression symptoms. It consists of two 4-point Likert items, ranging from 0 (not at all) to 3 (nearly every day). A score of 3 or greater suggests the potential for major depressive disorder. Previous research supports the measure's construct and criterion validity, discriminative validity, and test-retest reliability. (Kroenke et al., 2003; Lowe et al., 2005; Staples et al., 2019). The Overall Anxiety Severity and Impairment Scale (OASIS) was used to assess the frequency and intensity of anxiety symptoms. It consists of five 5-point Likert items, ranging from 0 (lowest value) to 4 (highest value), with higher scores indicating more frequent and severe anxiety. The OASIS has demonstrated strong psychometric properties, including good convergent validity and test-retest reliability. (Bragdon et al., 2016; Campbell et al., 2009; Norman et al., 2006). Other MindTrails studies have also found acceptable internal consistency for this measure (Ji et al., 2021; Larrazabal et al., 2024). Perceived Stress Scale 4 (PSS-4) was used to assess stress through four 5-point Likert items, ranging from 0 (never) to 4 (very often). A higher score indicates more stress. Previous research has found acceptable internal consistency and reliability for this measure (Ruisoto et al., 2020; Vallejo et al., 2018).” (p. 13)*

For reference, the citations are below:

Bragdon LB, Diefenbach GJ, Hannan S, Tolin DF. Psychometric properties of the Overall Anxiety Severity and Impairment Scale (OASIS) among psychiatric outpatients. J Affect Disord. 2016 Sep;201:112–5.

Campbell-Sills L, Norman SB, Craske MG, Sullivan G, Lang AJ, Chavira DA, et al. Validation of a brief measure of anxiety-related severity and impairment: The Overall Anxiety Severity and Impairment Scale (OASIS). J Affect Disord. 2009 Jan;112(1-3):92–101.

Ji JL, Baee S, Zhang D, Calicho-Mamani CP, Meyer MJ, Funk D, et al. Multi-session online interpretation bias training for anxiety in a community sample. Behav Res Ther. 2021 Jul;142:103864.

Kroenke K, Spitzer RL, Williams JB. The Patient Health Questionnaire-2: validity of a two-item depression screener. Med Care. 2003 Nov;41(11):1284–92. doi: 10.1097/01.MLR.0000093487.78664.3C. PMID: 14583691.

Larrazabal MA, Eberle JW, Vela A, Boukhechba M, Funk DH, Barnes LE, et al. Online cognitive bias modification for interpretation to reduce anxious thinking during the COVID-19 pandemic. Behav Res Ther. 2024 Feb 1;173:104463.

Löwe B, Kroenke K, Gräfe K. Detecting and monitoring depression with a two-item questionnaire (PHQ-2). J Psychosom Res. 2005 Feb;58(2):163–71.

Norman SB, Hami Cissell S, Means-Christensen AJ, Stein MB. Development and validation of an Overall Anxiety Severity and Impairment Scale (OASIS). Depress Anxiety. 2006;23(4):245–9.

Ruisoto P, López-Guerra VM, Paladines MB, Vaca SL, Cacho R. Psychometric properties of the three versions of the Perceived Stress Scale in Ecuador. Physiol Behav. 2020 Oct;224:113045.

Staples LG, Dear BF, Gandy M, Fogliati V, Fogliati R, Karin E, et al. Psychometric properties and clinical utility of brief measures of depression, anxiety, and general distress: The PHQ-2, GAD-2, and K-6. Gen Hosp Psychiatry. 2019 Jan;56:13–8.

Vallejo MA, Vallejo-Slocker L, Fernández-Abascal EG, Mañanes G. Determining factors for stress perception assessed with the Perceived Stress Scale (PSS-4) in Spanish and other European samples. Front Psychol. 2018;9:37. doi: 10.3389/fpsyg.2018.00037.

**Comment 19:** Provide a more robust justification for using a mixed-methods approach. Explain how the quantitative and qualitative data complement each other and how they will be integrated in the analysis and interpretation of results.

**Response 19:** Thank you for your suggestion. We have added the following comments on page 8 of the paper to provide a more comprehensive justification for our use of a mixed-methods approach:

“*Qualitative methods often provide greater depth in understanding, while quantitative methods are viewed as more objective and generalizable. (Lund, 2011). Mixed-methods analysis is often used to corroborate findings, elaborate on results, or generate new ideas (Rossman & Wilson, 1985). We employed a mixed-methods approach to corroborate findings and enhance the credibility of the results through triangulation, while also elaborating on the results to achieve a more comprehensive analysis of the data (Hussein, 2009). While qualitative analysis was the primary focus of the study, quantitative methods were used to complement, confirm, and expand upon the qualitative findings.” (p. 10)*

For reference, the citations are below:

Hussein A. The use of triangulation in social sciences research. J Comparative Soc Work. 2009 Apr 1;4(1):106–17.

Lund T. Combining qualitative and quantitative approaches: some arguments for mixed methods research. Scand J Educ Res. 2011 Apr;56(2):155–65. Available from: <https://doi.org/10.1080/00313831.2011.568674>

Rossman GB, Wilson BL. Numbers and words: combining quantitative and qualitative methods in a single large-scale evaluation study. Eval Rev. 1985 Oct;9(5):627–43.

**Comment 20:** Conduct a more thorough analysis of how responses varied across different demographic groups (e.g., students vs. faculty, different age groups). Present these findings in a clear, systematic manner, potentially using subheadings for each demographic category.

**Response 20:** Thank you for this suggestion. We agree that a more thorough analysis of how responses vary across the different groups will greatly enhance the paper. We have included tables showing responses to each survey question by group. Due to length, these tables are included in the appendix:

**S1 Tables: Results from Questionnaire by Group**

**Table 1: How helpful do you find Hoos Think Calmly for reducing your anxiety?**

| **Group** | **1 =**  **Not at all** | **2 = Slightly** | **3 = Somewhat** | **4 =**  **Mostly** | **5 =**  **Very** | **Mean ± SD** |
| --- | --- | --- | --- | --- | --- | --- |
| Faculty | 5  (33.33%) | 7   (46.67%) | 2  (13.33%) | 1  (6.67%) | 0  (0.00%) | 1.93 ± 0.88 |
| Staff | 5  (6.75%) | 18 (24.32%) | 36  (48.65%) | 11  (14.86%) | 3  (4.05%) | 2.85 ± 0.91 |
| Graduate students | 8  (10.53%) | 18 (23.68%) | 35  (46.05%) | 13  (17.11%) | 2  (2.63%) | 2.78 ± 0.95 |
| Undergraduates | 17  (28.33%) | 14 (23.33%) | 24  (40.00%) | 5  (8.33%) | 0  (0.00%) | 2.28 ± 0.98 |

*Percentages do not add up to 100 when “Prefer not to answer” was endorsed

**Table 2: How much could you relate to the stories presented during training sessions?**

| **Group** | **1 =**  **Not at all** | **2 = Slightly** | **3 = Somewhat** | **4 =**  **Mostly** | **5 =**  **Very** | **Mean ± SD** |
| --- | --- | --- | --- | --- | --- | --- |
| Faculty | 0  (0.00%) | 10 (76.92%) | 3   (23.07%) | 0  (0.00%) | 0  (0.00%) | 2.23 ± 0.44 |
| Staff | 2  (2.90%) | 14  (20.29%) | 29  (42.03%) | 16 (23.19%) | 7  (10.14%) | 3.18 ± 0.98 |
| Graduate students | 2  (2.82%) | 15 (21.13%) | 29  (40.85%) | 18 (25.35%) | 7  (9.86%) | 3.18 ± 0.98 |
| Undergraduates | 5  (8.77%) | 18 (31.58%) | 17  (29.82%) | 12 (21.05%) | 5  (8.77%) | 2.89 ± 1.11 |

*Percentages do not add up to 100 when “Prefer not to answer” was endorsed

**Table 3: How believable did you find the stories’ endings in terms of those outcomes happening in your life?**

| **Group** | **1 =**  **Not at all** | **2 = Slightly** | **3 = Somewhat** | **4 =**  **Mostly** | **5 =**  **Very** | **Mean ± SD** |
| --- | --- | --- | --- | --- | --- | --- |
| Faculty | 0  (0.00%) | 10 (76.92%) | 1  (7.69%) | 1  (7.69%) | 0  (0.00%) | 2.25 ± 0.62 |
| Staff | 1  (1.45%) | 16 (23.19%) | 23  (33.33%) | 19 (27.54%) | 8  (11.59%) | 3.25 ± 1.01 |
| Graduate students | 1  (1.30%) | 9  (12.68%) | 32  (45.07%) | 22 (30.99%) | 7  (9.86%) | 3.35 ± 0.88 |
| Undergraduates | 6  (10.53%) | 17 (29.82%) | 13  (22.81%) | 18 (31.58%) | 3  (5.26%) | 2.91 ± 1.12 |

*Percentages do not add up to 100 when “Prefer not to answer” was endorsed

**Table 4: I find the [length of the] training sessions to be:**

| **Group** | **1 =**  **Way too short** | **2 = Somewhat too short** | **3 = The right length** | **4 =**  **Somewhat too long** | **5 =**  **Way too long** | **Mean ± SD** |
| --- | --- | --- | --- | --- | --- | --- |
| Faculty | 0  (0.00%) | 1  (25.00%) | 0  (0.00%) | 1  (25.00%) | 1  (25.00%) | 3.67 ± 1.53 |
| Staff | 0  (0.00%) | 0  (0.00%) | 19 (61.29%) | 12  (38.71%) | 0  (0.00%) | 3.39 ± 0.50 |
| Graduate students | 0  (0.00%) | 0  (1.09%) | 17 (50.00%) | 13  (44.57%) | 0  (2.17%) | 3.43 ± 0.50 |
| Undergraduates | 0  (0.00%) | 0  (0.00%) | 10 (38.46%) | 15  (57.69%) | 1  (3.85%) | 3.65 ± 0.56 |

*Percentages do not add up to 100 when “Prefer not to answer” was endorsed

Similarly, we analyzed the interview data by group, examining the breakdown by code, and included the notable findings throughout the Results section under the relevant subheadings.

Under “Application of New Coping Strategies”:

*“Notably, of these 11 participants, 8 of them were graduate students (73%), 2 were undergraduate students (18%), 1 was staff (9%), and none were faculty.” (p. 18)*

Under “Relatability”:

*“Notably, of these 11 participants that found the program relatable, 8 of them were graduate students (73%), 2 were undergraduate students (18%), 1 was staff (9%), and none were faculty.” (p. 19)*

In the Discussion, we compared these qualitative and quantitative findings:

*“Data was also analyzed by demographic/university role breakdown. A triangulation-based framework was adopted to compare the quantitative results with the corresponding qualitative data. When examining the perceived length of the program based on both quantitative and qualitative data, no demographic group disproportionately favored one response over the other groups. Instead, the general consensus was split between those who found the program to be the right length and those who considered it too long, as indicated in both the qualitative and quantitative findings. In regards to the program effectiveness, quantitative findings indicate that faculty tend to rate the program less positively than the other groups, finding it the least helpful, relatable, and believable. In contrast, staff and graduate students generally rate the program more positively, being more likely to find it helpful, relatable, and believable. Undergraduates fall somewhere in between (see S1 Tables). Qualitative data align with these quantitative findings, as all faculty interviewees described the program as unrelatable, and none reported an increase in flexible thinking skills. Meanwhile, the majority of graduate interviewees (8/10, 80%) found the scenarios relatable and noted an increase in flexible thinking skills. These findings suggest a relationship between the relatability of the scenarios and the perceived effectiveness of the program. Overall, both quantitative and qualitative results indicate that the current version of HTC may be best suited for graduate students, and further work is needed to improve the program for faculty.” (p. 28-29)*

**Comment 21:** Provide a more comprehensive synthesis of the mixed-methods results. Develop a framework or model that illustrates how the quantitative and qualitative findings complement each other and contribute to a holistic understanding of user experiences with HTC.

**Response 21:** We now include a more in-depth analysis of the quantitative and qualitative findings by demographic group and have clarified our triangulation-based framework to directly compare these quantitative results with the corresponding qualitative findings (see above).

**Comment 22:** Elaborate on the potential benefits and challenges of increasing app personalization and customization. Discuss the trade-offs between user preferences and maintaining intervention fidelity. Provide specific recommendations for balancing these competing demands in future iterations of HTC and similar DMHIs.

**Response 22:** Thank you for your feedback. We have updated this section of the discussion.

*“Although allowing participants to customize the type and length of training sessions may increase app engagement and user satisfaction, there is a concern that increasing customizability of training scenario type and length may sacrifice program effectiveness (e.g., if users reliably select such brief training periods that the dose is insufficient to reduce their engrained anxious thinking). Additionally, other research has found that too many customizable features may be burdensome and frustrating for people with motivational and cognitive challenges (Zhang et al., 2021). Instead, a focus on low-burden customization can enhance user autonomy and engagement with mental health interventions. For instance, studies indicate that daily checklists or progress tracking serve as effective, low-burden customizations that can enhance a sense of control and satisfaction (Ayobi et al., 2018; Choe et al., 2017).*

*To balance customization, user engagement, and app efficacy, it’s essential to implement guided customization that provides users with a limited set of meaningful options, enhancing autonomy while preserving app integrity. In developing a DMHI, involving users in the design process is crucial to identify the most relevant customizable features for that demographic group. Additionally, as customization options are introduced, ongoing assessment of user satisfaction, engagement, and program efficacy is vital for understanding the trade-offs involved.” (32-33)*

For reference, the citations are below:

Ayobi A, Sonne T, Marshall P, Cox AL. Flexible and mindful self-tracking: design implications from paper bullet journals. In: Proceedings of the 2018 CHI Conference on Human Factors in Computing Systems (CHI '18). New York, NY, USA: Association for Computing Machinery; 2018. p. Paper 28, 1–14. Available from: <https://doi.org/10.1145/3173574.3173602>

Choe EK, Abdullah S, Rabbi M, Thomaz E, Epstein DA, Cordeiro F, et al. Semi-automated tracking: a balanced approach for self-monitoring applications. IEEE Pervasive Comput. 2017 Jan;16(1):74–84.

Zhang R, Ringland KE, Paan M, Mohr DC, Reddy M. Designing for emotional well-being: integrating persuasion and customization into mental health technologies. In: Proceedings of the 2021 CHI Conference on Human Factors in Computing Systems (CHI '21). New York, NY, USA: Association for Computing Machinery; 2021. p. Article 542, 1–13. Available from: <https://doi.org/10.1145/3411764.3445771>

**Comment 23:** Offer a more in-depth examination of the barriers to app engagement identified in the study. Categorize these barriers (e.g., technical, personal, contextual) and discuss their implications for app design, implementation, and user support strategies.

**Response 23:** Thank you for these suggestions to better organize the Discussion. We have rearranged the content to identify and elaborate on the barriers to engagement within a single section.

*“Three main barriers to engaging with the platform were identified: contextual, personal, and technical. Contextual barriers arose from participants' lack of understanding of the intervention’s rationale and how to engage with the program, hindering their ability to use it effectively. To address this, providing clear instructions on program expectations and guidelines for recommended use in multiple locations may enhance user engagement, as this information often appears to be overlooked or skimmed. Consistent with prior qualitative research, insufficient task explanations have been shown to deter users from continuing with digital health interventions [65]. Therefore, it is essential to present instructions clearly, and it may help to use various mediums [66]. HTC incorporates text-based and video instructions; to enhance clarity regarding task expectations, our users specifically suggest implementing a daily progress tracker, which could include gamification elements that might increase user motivation and engagement [67] while also clarifying what tasks need to be completed each day.*

*Personal barriers to engaging with the app included individuals citing forgetfulness as a reason for not participating in the program. This issue is directly linked to the technical barrier of notification problems, which is particularly important given half of the interviewees reported struggling with forgetfulness. Many participants indicated that they used the program specifically when reminded and appreciated the ability to schedule notifications at specific times. However, those who encountered issues with notifications stated that they only appeared when the app was open or running in the background. If the app was completely closed, reminders did not come through. This problem seemed specific to certain app versions and/or phone operating systems. Given that forgetfulness is a significant barrier to using the app, these technical issues likely impacted overall user engagement. To enhance notification customization and address both personal and technical barriers, we recommend offering users a range of notification delivery options, including push notifications, text messages, emails, or calendar reminders.” (p. 30-31)*

**Comment 24:**Provide a more comprehensive analysis of notification preferences and their impact on user engagement. Discuss the potential benefits and drawbacks of different notification strategies, and offer specific recommendations for optimizing notification systems in DMHIs.

**Response 24:** We have expanded on our analysis of notification preferences in the discussion. Please see reviewer #1 response 4, and reviewer #3 response 23 above.

**Comment 25:** Offer a more critical examination of the study's limitations. Discuss how these limitations might have influenced the findings and provide specific recommendations for addressing these limitations in future research.

**Response 25:** We have expanded the study’s limitations to include a more thorough examination of sample size, demographics, and engagement.

“*Specifically, we did not achieve the desired sample size for faculty, and this underrepresentation may limit our ability to draw robust conclusions. To address this, future research should prioritize targeted recruitment efforts for faculty, as they were identified as the most challenging demographic to engage. Additionally, most participants identified as White, non-Hispanic, and female, which means our results may not accurately represent the experiences of individuals from other racial, ethnic, and gender backgrounds.*

*Additional limitations include factors that may impact engagement, such as technical issues and study compensation. Participants reported technical issues, specifically regarding notifications, which hindered their ability to receive app reminders and likely affected their engagement. To address this limitation, future research should consider expanding notification delivery options to ensure participants receive alerts in a more accessible and effective format. Additionally, while participants received a $50 compensation for their involvement, we cannot determine how engagement might have differed without this incentive.” (p. 35)*

**Comment 26:** Provide a more comprehensive analysis of the potential clinical implications of these findings. Discuss how the results might inform clinical practice, particularly in terms of recommending and supporting the use of DMHIs like HTC.

**Response 26:** Thank you for this suggestion. We have expanded on the potential clinical implications of these findings.

*“Our findings indicate that HTC is perceived as somewhat acceptable and effective at reducing stress and anxiety, with higher rates of perceived effectiveness among graduate students. Further research is needed to explore the general acceptability of DMHIs across demographic groups. However, the results suggest potential clinical implications, including the possibility of adding HTC to other forms of mental health care to support users in daily life (e.g., providing help managing stressful situations in between therapy sessions or medication check-ins). Alternatively, for users with minimal symptoms, using HTC may help manage symptoms so they do not escalate and reduce the need for (resource-intensive) care with a mental health professional. Additionally, HTC could be a valuable addition to support patients who are on waitlists so they have some support and symptoms do not worsen. Each of these possibilities requires further empirical evaluation, but given the difficulties accessing care and widespread need, DMHIs hold promise for increasing access to care.*

*At the same time, barriers such as forgetfulness and a lack of understanding regarding how to engage with the training sessions or the purpose of the intervention were noted. To address these challenges and optimize clinical impact, we recommend providing support and establishing regular check-ins when implementing DMHIs in a clinical setting. These check-ins could assess how users are interacting with the app, address any barriers they encounter, and offer encouragement or support as needed to help sustain user motivation and adherence to the program.” (p. 33-34)*

**Comment 27:** Provide a more impactful conclusion that synthesizes the key findings, their implications, and future directions. Offer a clear and compelling vision for the future development and implementation of HTC and similar DMHIs.

**Response 27:** We have revised the conclusion to provide a clearer and more compelling summary of the paper.

*“This study used mixed methods to explore users' experiences with the HTC platform, collecting feedback on its acceptability and suggestions for improvements, which could inform design of DMHIs more broadly. Qualitative data were gathered through user interviews, while quantitative data were collected via user experience questionnaires, specifically analyzing the program's effectiveness, relatability of scenarios, and opinions on session length. A triangulation-based framework was employed to compare the quantitative and qualitative findings, mostly demonstrating agreement between the methods. Faculty rated the program less positively than other groups, while graduate students found it the most effective and relatable. Undergraduate students and staff rated it moderately positive with somewhat more mixed results. The results highlighted a gap in content relatability for some users, which may have influenced their perceptions of the app's efficacy. Recommendations for future iterations include clearer introductory content, enhanced personalization of training scenarios, and improved notification systems to address user engagement barriers. Overall, while HTC showed promise in reducing stress and anxiety, further research is needed to understand its acceptability across diverse demographic groups and to refine the program for optimal effectiveness.” (p. 36)*
